# Left atrial acceleration factor as a magnetic resonance 4D flow measure of mean pulmonary artery wedge pressure in pulmonary hypertension

**DOI:** 10.3389/fcvm.2022.972142

**Published:** 2022-08-03

**Authors:** Gert Reiter, Gabor Kovacs, Clemens Reiter, Albrecht Schmidt, Michael Fuchsjäger, Horst Olschewski, Ursula Reiter

**Affiliations:** ^1^Research & Development, Siemens Healthcare Diagnostics GmbH, Graz, Austria; ^2^Division of General Radiology, Department of Radiology, Medical University of Graz, Austria; ^3^Division of Pulmonology, Department of Internal Medicine, Medical University of Graz, Austria; ^4^Ludwig Boltzmann Institute for Lung Vascular Research Graz, Austria; ^5^Division of Cardiology, Department of Internal Medicine, Medical University of Graz, Austria

**Keywords:** right heart catheterization (RHC), pulmonary hypertension, pulmonary artery wedge pressure, cardiac magnetic resonance (CMR) imaging, 4D flow

## Abstract

**Background:**

Mean pulmonary artery wedge pressure (PAWP) represents a right heart catheter (RHC) surrogate measure for mean left atrial (LA) pressure and is crucial for the clinical classification of pulmonary hypertension (PH). Hypothesizing that PAWP is related to acceleration of blood throughout the LA, we investigated whether an adequately introduced LA acceleration factor derived from magnetic resonance (MR) four-dimensional (4D) flow imaging could provide an estimate of PAWP in patients with known or suspected PH.

**Methods:**

LA 4D flow data of 62 patients with known or suspected PH who underwent RHC and near-term 1.5 T cardiac MR (ClinicalTrials.gov identifier: NCT00575692) were retrospectively analyzed. Early diastolic LA peak outflow velocity (*v*_E_) as well as systolic (*v*_S_) and early diastolic (*v*_D_) LA peak inflow velocities were determined with prototype software to calculate the LA acceleration factor (α) defined as α = *v*_E_/[(*v*_S_ + *v*_D_)/2]. Correlation, regression and Bland-Altman analysis were employed to investigate the relationship between α and PAWP, α-based diagnosis of elevated PAWP (>15 mmHg) was analyzed by receiver operating characteristic curve analysis.

**Results:**

α correlated very strongly with PAWP (*r* = 0.94). Standard deviation of differences between RHC-derived PAWP and PAWP estimated from linear regression model (α = 0.61 + 0.10·PAWP) was 2.0 mmHg. Employing the linear-regression-derived cut-off α = 2.10, the α-based diagnosis of elevated PAWP revealed the area under the curve 0.97 with sensitivity/specificity 93%/92%.

**Conclusions:**

The very close relationship between the LA acceleration factor α and RHC-derived PAWP suggests α as potential non-invasive parameter for the estimation of PAWP and the distinction between pre- and post-capillary PH.

## Introduction

Mean pulmonary artery wedge pressure (PAWP) represents a surrogate for mean left atrial (LA) pressure (1, 2), and its assessment by right heart catheterization (RHC) is crucial in the clinical classification of pulmonary hypertension (PH). A cut-off value of PAWP = 15 mmHg is employed to differentiate between pre- and post-capillary PH. In addition, the difference between the mean pulmonary arterial pressure (mPAP) and PAWP defines the transpulmonary pressure gradient, which enters in the calculation of pulmonary vascular resistance (3, 4). While magnetic resonance (MR) four-dimensional (4D) flow imaging has been shown to allow for an accurate estimation of elevated mPAP and the diagnosis of PH from the duration of vortical blood flow along the pulmonary artery (5, 6), a non-invasive 4D flow-based correlate to PAWP is lacking.

If there is one, then a flow-based correlate for PAWP is likely to be encoded in LA blood flow patterns. MR 4D flow imaging of the LA has mainly been employed to study vortical blood flow and stasis within the LA (7–9). The LA pressure represents both the driving force of transmitral outflow from the LA and the decelerating force for pulmonary venous inflow into the LA. Consequently, it might be expected that higher mean LA pressure or PAWP could be related to lower LA inflow velocities and higher LA outflow velocities or—summarizing both effects—to a higher “acceleration” of blood from LA inflow to outflow.

We therefore hypothesized that PAWP should correlate closely with a LA acceleration factor—defined as an adequate ratio of 4D flow-derived LA peak outflow to peak inflow velocities. The purpose of the present study was to investigate the relationship between the LA acceleration factor and PAWP in patients with known or suspected PH.

## Materials and methods

### Study population

Between June 2006 and May 2014 240 patients with known or suspected PH, aged above 18 years and without known MR contraindications were recruited for near-term cardiac MR and 4D flow imaging after successful RHC. This prospective study (ClinicalTrials.gov identifier, NCT00575692) complied with the Declaration of Helsinki, was approved by the local ethical review board and all subjects gave written informed consent. 4D flow in the right ventricular outflow tract of the subjects was explored previously (5, 6, 10, 11). In the current study we retrospectively analyzed data from those 70 patients where LA 4D flow containing the orifices of the right and left inferior pulmonary veins as well as the mitral valve was acquired. To minimize the probability of relevant changes in disease state, eight patients for whom a delay of one month or more occurred between RHC and MR imaging were excluded from analysis. Mean delay between RHC and MR imaging for the remaining 62 patients was 7 ± 10 days; the median delay was one day. No changes in drug treatment occurred between RHC and MR imaging.

### Right heart catheterization

RHC was performed during free breathing in the supine position with a 7F quadruple-lumen, balloon-tipped, flow-directed Swan-Ganz catheter (Baxter Healthcare Corporation, Irvine, CA, USA) using the transjugular approach. Zero reference level was set to anterior axillary line. All intrathoracic pressures—including PAWP—were averaged over 3–4 respiratory cycles during normal respiration. No triggering on any respirator or cardiac waves was employed (4, 12). RHC parameters obtained included mPAP, systolic and diastolic pulmonary arterial pressure (sPAP and dPAP, respectively), PAWP, TPG and PVR with the cardiac output measured by thermodilution. Moreover, systolic and diastolic systemic blood pressure (sBP and dBP, respectively) were measured by sphygmomanometer. Following the current guidelines (4) thresholds of mPAP ≥ 25 mmHg and of PAWP > 15 mmHg were employed to diagnose PH and post-capillary PH, respectively.

### MR imaging

Electrocardiographically (ECG)-gated cardiac MR imaging, including functional imaging and 4D flow imaging of the left atrium, was performed on a 1.5 T MR scanner (MAGNETOM Sonata, Siemens Healthcare, Erlangen, Germany) using a 6-channel cardiac-array coil together with a spine array coil. Subjects were investigated in the supine position.

For functional assessment, retrospectively ECG-gated, balanced steady state free precession (bSSFP) cine series were obtained in 2-chamber and 4-chamber views as well as in contiguous gapless short-axis slices covering the ventricles. Imaging parameters were as follows: temporal resolution, 32–41 ms in long-axis and 48–54 ms in short-axis view; interpolated cardiac phases, 30; echo time, 1.2 ms; flip angle, 60°; bandwidth, 930 Hz/pixel; voxel size, 1.7–1.9 × 1.4 × 6.0 mm^3^ in long-axis and 2.2 × 1.4 × 8.0 mm^3^ in short-axis view; imaging time per slice, 12–15 heart beats in long-axis and 6–8 heart beats in short-axis view.

4D flow imaging data were acquired employing a retrospectively ECG-gated, segmented, two-dimensional (2D) spoiled gradient-echo-based cine phase-contrast sequence with three-directional velocity encoding by a simple four-point velocity encoding scheme. The LA was covered by gapless slices in 4-chamber orientation containing the orifices of the right and left inferior pulmonary veins as well as the mitral valve. Velocity encoding (VENC) was set to 90 cm/s in all directions and was adapted—if necessary—to prevent aliasing in regions close to the mitral valve. Further protocol parameters were as follows: temporal resolution, 89 ms; interpolated cardiac phases, 20; echo time, 4.1 ms; flip angle, 15°; bandwidth, 455 Hz/pixel; GRAPPA (generalized auto-calibrating partially parallel acquisition) factor, 2; voxel size, 2.4 × 1.8 × 6.0 mm^3^; imaging time per slice, 22–24 heart beats.

Functional and 4D flow imaging were performed during multiple breath holding. For patients who were unable to hold their breath, three-fold averaging was used to suppress breathing artifacts.

### MR image analysis

Quantification of left ventricular (LV) and right ventricular (RV) end-diastolic volume (EDV), end-systolic volume (ESV), ejection fraction (EF), stroke volume (SV) and muscle mass (MM) was performed with standard software (Argus, Siemens Healthcare, Erlangen, Germany) by manual segmentation of myocardial end-diastolic and end-systolic epicardial and endocardial borders in the stack of bSSFP short-axis images including papillary muscles and trabeculae to the myocardium. Cardiac output (CO) was calculated from SV multiplied by the average heart rate during the short-axis scans. Additionally to ventricular volumetry, maximal LA volume (LAV_max_) was calculated from cine bSSFP 2-chamber and 4-chamber images employing the bi-planar area-length method (13). All non-relative volumetric cardiac parameters were normalized to body surface area (BSA) which is indicated with “I.”

**Figure 1:**
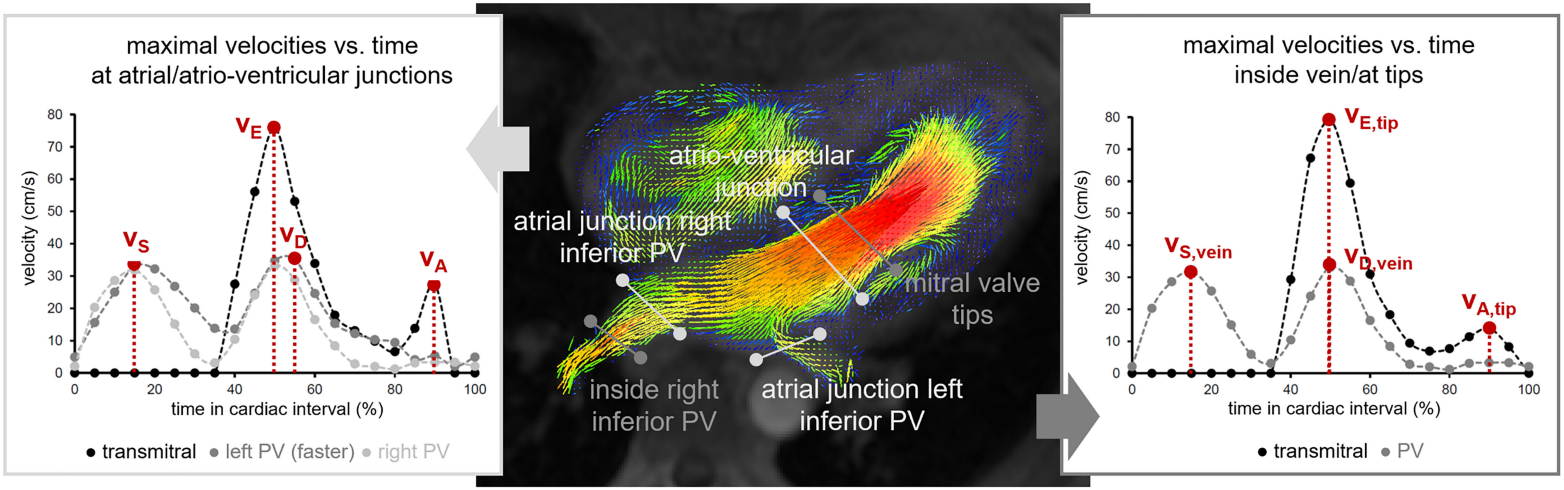
Illustration of the determination of LA peak in- and outflow velocities. Measurement cut planes are indicated on an early diastolic magnitude image with color-encoded vector plot of the three-dimensional velocity field. PV pulmonary vein; *v*_S_, systolic LA peak inflow velocity; *v*_D_, diastolic LA peak inflow velocity; *v*_E_, early diastolic LA peak outflow velocity; *v*_A_, late diastolic LA peak outflow velocity; *v*_S, vein_, systolic peak velocity determined in the pulmonary vein; *v*_D, vein_, diastolic peak velocity determined in the pulmonary vein; *v*_E, tip_, early diastolic peak velocity determined at the level of the mitral valve tips; *v*_A, tip_, late diastolic peak velocity determined at the level of the mitral valve tips.

Time courses of maximal 3-dimensional (3D) velocities across the time-varying planes of the atrio-ventricular junction and the atrial junctions of the left and right inferior pulmonary veins were determined from 4D flow data employing dedicated prototype software (4D Flow, Siemens Healthcare, Erlangen, Germany). Early diastolic (*v*_E_) and late diastolic (*v*_A_) LA peak outflow velocities were defined as early and late diastolic peaks of the transmitral maximal velocity-time curve; systolic (*v*_S_) and diastolic (*v*_D_) LA peak inflow velocities were defined as systolic and early diastolic peaks of the left or right pulmonary venous maximal velocity-time curve, whichever exhibited higher velocities (Figure 1). The LA acceleration factor (α) was defined as the following ratio of a specific linear combination of *v*_E_ and *v*_A_ to a specific linear combination of *v*_S_ and *v*_D_:


$$\begin{array}{l} \alpha \;=\;\frac{v_{E}}{\frac{\left(v_{S}\;+\;v_{D}\right)}{2}} \end{array}$$

To evaluate the impact of the location of velocity measurements on α, peak LA in- and outflow velocities were additionally determined from spatially fixed planes at the level of the mitral valve tips in early diastole and in the pulmonary vein with the fastest velocity 1–2 cm from the orifices (Figure 1). Maximum velocities are denoted by *v*_E, tip_, *v*_A, tip_, *v*_S, vein_ and *v*_D, vein_, the corresponding LA acceleration factors by α_tip_, α_vein_ and α_tip/vein_, respectively.

### Statistical analysis

Statistical analysis was performed using NCSS software (NCSS 11 Statistical Software (2016). NCSS, LLC. Kaysville, Utah, USA) employing a significance level of 0.05 for statistical tests. Mean values are given together with standard deviations and diagnostic measures are specified together with 95%-confidence intervals. The nomenclature 0.3–0.5 = weak, 0.5–0.7 = moderate, 0.7–0.9 = strong, and 0.9–1.0 = very strong is employed for classifying the strength of a correlation by the Pearson's correlation coefficient (*r*).

Demographic, RHC hemodynamic, volumetric cardiac and in- and outflow peak velocity parameters in the non-PH, the pre-capillary and the post-capillary PH groups were compared by one-way analysis of variance (ANOVA) and Tukey-Kramer multiple comparison procedure. Relationships between these parameters were investigated by means of correlation and linear regression analysis. The dependency of slopes and intercepts of the linear regression models on binary variables was analyzed by adding them (with and without interaction) separately to the respective linear regression model and performing partial *F*-tests. Similarly, bilinear regressions on PAWP and continuous demographic, RHC hemodynamic as well as volumetric cardiac parameters were introduced and analyzed by partial *F*-tests to determine if the latter parameters could explain a significant amount of residual variation in the corresponding linear regression model on PAWP.

Non-linear regression of PAWP on *v*_E_, *v*_A_, *v*_S_ and *v*_D_ was employed to motivate the definition of the LA acceleration factor α as linear correlate to PAWP among all general ratios (α_general_) of linear combinations of LA peak outflow velocities to linear combinations of LA peak inflow velocities: Up to a multiplicative constant such a general ratio can be written as


$$\begin{array}{l} \alpha_{general}\;=\;\frac{v_{E+A}\;+\;{\;c}_{out}\cdot v_{E-A}}{v_{S+D}\;+\;{\;c}_{in}\cdot v_{S-D}}, \end{array}$$

where *c*_out_ and *c*_in_ are constants and where *v*_E+A_, *v*_E−A_, *v*_S+D_, and *v*_S−D_ represent short notations for (*v*_E_ + *v*_A_)/2, (*v*_E_ – *v*_A_)/2, (*v*_S_ + *v*_D_)/2, and (*v*_S_ – *v*_D_)/2, respectively. A specific choice of α_general_ was interpreted as optimal if the choices for c_out_ and c_in_ were contained within the large sample 95%-confidence intervals of the non-linear regression of PAWP on *v*_E_, *v*_A_, *v*_S_ and *v*_D_.

The linear regression equation of α on PAWP was inverted to derive calculated mean pulmonary arterial wedge pressure values PAWP_calc_ from α and to compare PAWP_calc_ and PAWP by Bland-Altman analysis. The diagnostic performance of α in predicting PAWP > 15 mmHg was investigated by means of receiver operating characteristic curve (ROC) analysis.

One-sample *t*-test was employed to test if differences of quantities from velocity measurements at different locations differed from zero. Pearson correlation coefficients between PAWP and LA acceleration factors derived from velocity measurements at different locations were compared by means of Williams-Hotelling test.

## Results

PH was diagnosed in 34 of the analyzed 62 patients; nine patients demonstrated mPAP between 21 and 24 mmHg, one of them with PAWP > 15 mmHg. Nineteen patients had normal mPAP <21 mmHg. Thirteen PH patients with PAWP > 15 mmHg were classified as having post-capillary PH. The remaining 21 pre-capillary PH patients suffered from pulmonary arterial hypertension (*n* = 15), PH due to lung diseases (*n* = 2), chronic thromboembolic PH (*n* = 2) and PH with unclear multifactorial mechanisms (*n* = 2). Demographic, RHC-derived hemodynamic and volumetric cardiac parameters of the study population derived from RHC are summarized in Table 1. Among all volumetric parameters LAVI_max_ demonstrated the strongest correlation to PAWP (*r* = 0.49). After omission of one severe outlier in the pre-capillary PH group the correlation increased to *r* = 0.60; the significance of differences in LAVI_max_ between non-PH, pre-capillary PH and post-capillary PH subjects remained unaltered.

**Table 1:** Demographic, RHC-derived hemodynamic and volumetric cardiac data of the study population.

**Parameter**	**All**	**Non-PH**	**Pre-capillary PH**	**Post-capillary PH**	***p*-value**
Demographic data					
Number	62	28	21	13	
Male/female	16/46	4/24	7/14	5/8	
Age (years)	63 ± 12	59 ± 12	64 ± 12	68 ± 9	0.0727
BSA (m^2^)	1.8 ± 0.2	1.8 ± 0.2	1.9 ± 0.2	1.9 ± 0.2	0.1501
RHC data					
Heart rate (min^−1^)	73 ± 13	70 ± 10	76 ± 15	75 ± 12	0.1832
mPAP (mmHg)	29 ± 14	17 ± 4^1, 2^	37 ± 10^0^	40 ± 15^0^	<0.0001
sPAP (mmHg)	45 ± 22	27 ± 7^1, 2^	61 ± 16^0^	55 ± 25^0^	<0.0001
dPAP (mmHg)	18 ± 9	11 ± 3^1, 2^	24 ± 8^0^	23 ± 7^0^	<0.0001
PAWP (mmHg)	11 ± 5	8 ± 4^2^	10 ± 4^2^	18 ± 3^0, 1^	<0.0001
TPG (mmHg)	18 ± 13	9 ± 4^1, 2^	28 ± 11^0^	22 ± 14^0^	<0.0001
PVR (Wood units)	3.4 ± 2.5	1.8 ± 0.8^1, 2^	5.2 ± 2.4^0^	4.1 ± 2.9^0^	<0.0001
sBP (mmHg)	128 ± 20	124 ± 21^2^	124 ± 13^2^	142 ± 23^0, 1^	0.0125
dBP (mmHg)	63 ± 10	63 ± 10	61 ± 9	66 ± 12	0.3742
Volumetric cardiac data					
Heart rate (min^−1^)	68 ± 12	66 ± 11	70 ± 14	69 ± 11	0.4400
LVEF (%)	66 ± 9	66 ± 10	66 ± 8	64 ± 11	0.8310
LVEDVI (ml·m^−2^)	63 ± 16	63 ± 17	60 ± 15	67 ± 17	0.4907
LVESVI (ml·m^−2^)	22 ± 9	22 ± 10	21 ± 9	24 ± 10	0.6205
LVSVI (ml·m^−2^)	41 ± 11	41 ± 11	40 ± 10	43 ± 15	0.6628
LVCI (l·min^−1^·m^−2^)	2.8 ± 0.8	2.7 ± 0.8	2.7 ± 0.8	2.9 ± 0.8	0.6161
LVMMI (g·m^−2^)	55 ± 14	51 ± 10	56 ± 14	60 ± 19	0.1071
RVEF (%)	52 ± 10	56 ± 8^1^	49 ± 10^0^	49 ± 11	0.0150
RVEDVI (ml·m^−2^)	92 ± 42	75 ± 29^1^	107 ± 50^0^	104 ± 43	0.0165
RVESVI (ml·m^−2^)	46 ± 27	34 ± 19^1, 2^	56 ± 30^0^	56 ± 30^0^	0.0065
RVSVI (ml·m^−2^)	46 ± 19	41 ± 13	51 ± 25	48 ± 17	0.1777
RVCI (l·min^−1^·m^−2^)	3.1 ± 1.2	2.7 ± 0.9	3.5 ± 1.4	3.3 ± 1.3	0.0615
RVMMI (g·m^−2^)	38 ± 18	28 ± 11^1, 2^	49 ± 21^0^	43 ± 15^0^	0.0001
LAVI_max_ (ml·m^−2^)	54 ± 33	41 ± 23^2^	61 ± 46	70 ± 19^0^	0.0155

*Parameters are given as means ± standard deviations. p-value refers to ANOVA comparison of mean values for non-PH, pre-capillary and post-capillary PH subjects. Superscripts 0, 1 and 2 indicate significant differences from non-PH, pre-capillary PH and post-capillary PH groups, respectively.*

*PH, pulmonary hypertension; RHC, right heart catheter; BSA, body surface area; mPAP, mean pulmonary arterial pressure; sPAP, systolic pulmonary arterial pressure; dPAP, diastolic pulmonary arterial pressure; PAWP, mean pulmonary artery wedge pressure; TPG, transpulmonary pressure gradient; PVR, pulmonary vascular resistance; sBP, systolic systemic blood pressure; dBP, diastolic systemic blood pressure; LV, left ventricle; RV, right ventricle; EF ejection fraction; EDVI, normalized end-diastolic volume; ESVI, normalized end-systolic volume; SVI, normalized stroke volume; CI, cardiac index; MMI, normalized muscle mass; LAVI_max_, normalized maximal left atrial volume*.

### LA peak in- and outflow velocities

LA peak inflow velocities were determined from the left inferior pulmonary vein's inflow in 25 subjects and from the right inferior pulmonary vein's inflow in 37 subjects. *v*_A_ could not be specified in six of the 62 patients because a biphasic transmitral outflow profile was lacking. Mean LA peak in- and outflow velocities in non-PH, pre-capillary PH and post-capillary PH subjects are summarized in Table 2. All LA peak in- and outflow velocities correlated significantly with each other except *v*_S_ with *v*_E_ and *v*_D_ with *v*_A_ (*r* = 0.38, 0.58, 0.39, and 0.40 for the correlations of *v*_S_ with *v*_D_, *v*_S_ with *v*_A_, *v*_D_ with *v*_E_ and *v*_E_ with *v*_A_, respectively).

**Table 2:** LA peak in- and outflow velocities.

**Parameter**	**All**	**Non-PH**	**Pre-capillary PH**	**Post-capillary PH**	***p*-value**
*v*_S_ (cm·s^−1^)	34 ± 13	39 ± 11^2^	32 ± 10	24 ± 14^0^	0.0006
*v*_D_ (cm·s^−1^)	33 ± 9	33 ± 9	33 ± 9	31 ± 9	0.7291
*v*_E_ (cm·s^−1^)	54 ± 19	53 ± 19^2^	47 ± 10^2^	68 ± 23^0, 1^	0.0048
*v*_A_ (cm·s^−1^)	39 ± 16	38 ± 10	39 ± 16	42 ± 27	0.7966

*Parameters are given as means ± standard deviations. p-value refers to ANOVA comparison of mean values for non-PH, pre-capillary and post-capillary PH subjects. Superscripts 0, 1 and 2 indicate significant differences in comparison to non-PH, pre-capillary PH and post-capillary PH groups, respectively.*

*PH, pulmonary hypertension; RHC, right heart catheter; v_S_, systolic LA peak inflow velocity; v_D_, diastolic LA peak inflow velocity; v_E_, early diastolic LA peak outflow velocity; v_A_, late diastolic LA peak outflow velocity*.

**Figure 2:**
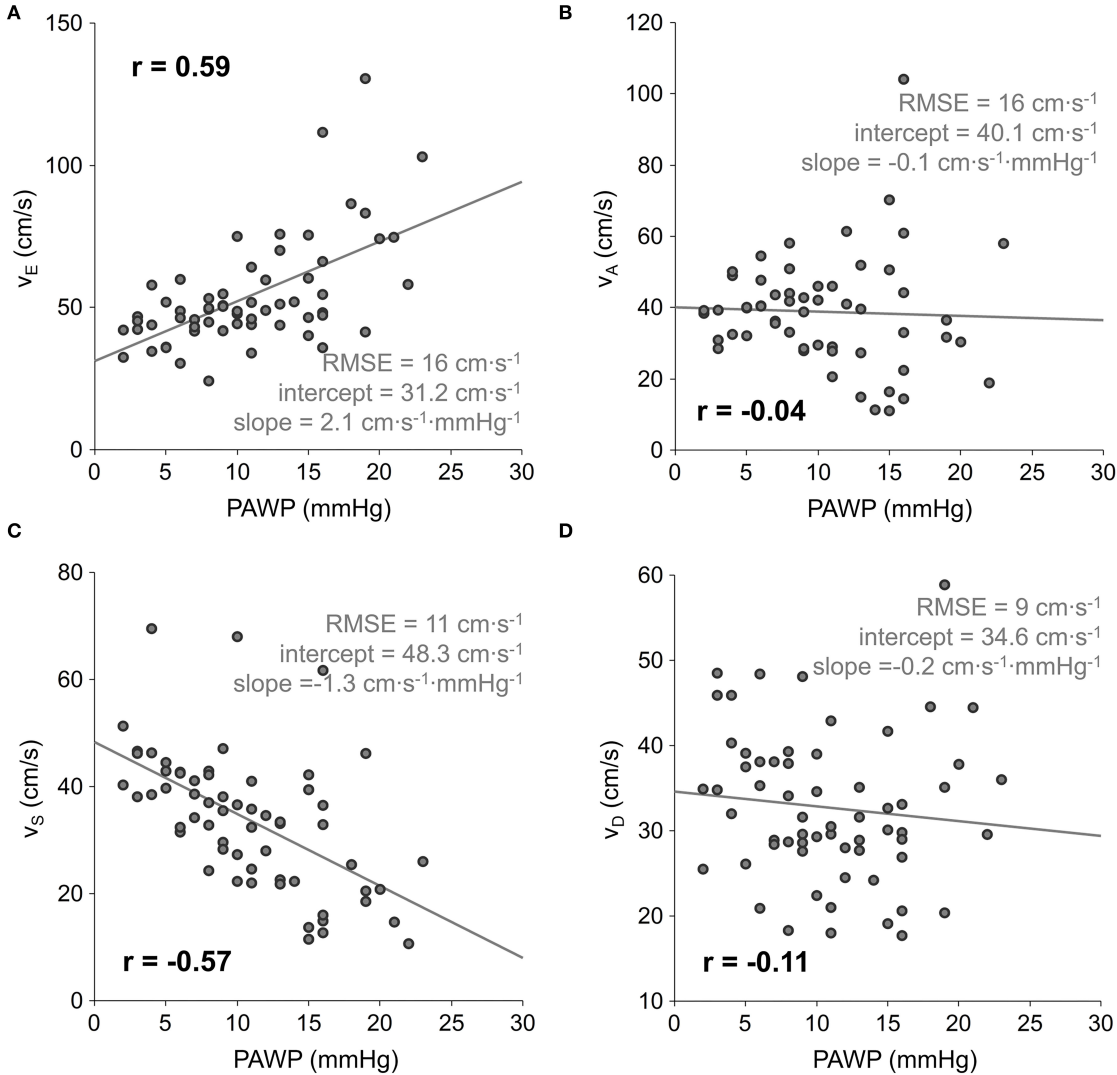
Scatter plots and linear regressions of early diastolic LA peak outflow *v*_E_ **(A)**, late diastolic LA peak outflow *v*_D_ **(B)**, systolic LA peak inflow *v*_S_ **(C)**, and early diastolic LA peak inflow *v*_D_ **(D)** velocity on PAWP. PAWP, mean pulmonary artery wedge pressure; r, correlation coefficient; RMSE, root-mean-square error.

*v*_S_ correlated moderately negatively (*r* = −0.57) and *v*_E_ moderately positively (*r* = 0.59) with PAWP, whereas *v*_D_ and *v*_A_ did not show significant correlations with PAWP. The linear regression results of LA peak in- and outflow velocities are shown in Figure 2. Additionally, all LA peak in- and outflow velocities exhibited weak to moderate, but significant univariate correlations with demographic, RHC hemodynamic and volumetric cardiac parameters, which are specified in Table 3. Notably, none of the RHC parameters remained a significant predictor of *v*_S_ when added to PAWP.

**Table 3:** Significant correlations of LA peak in- and outflow velocities with demographic, RHC-derived hemodynamic and MR-derived volumetric cardiac parameters.

**Para-meter**	**Demo-graphic**	**RHC**	**Volumetric LV**	**Volumetric RV**	**Volumetric LA**
*v*_S_	age (−0.44)	PAWP (−0.57) mPAP (−0.42) dPAP (−0.31) sPAP (−0.30)	LVEF (0.27) LVSVI (0.25)	RVEF (0.40) RVESVI (−0.37) RVEDVI (−0.28)	LAVI_max_ (−0.41)
*v*_D_	age (−0.42)	sBP (−0.44) dBP (−0.29)	LVCI (0.39) LVSVI (0.28)		
*v*_E_		PAWP (0.59)	LVCI (0.56) LVSVI (0.53) LVEDVI (0.42)		
*v*_A_		sBP (0.30)	LVEF (0.42) LVESVI (−0.31) LVCI (0.27) LVSVI (0.25)	RVEF (0.35) RVESVI (−0.33) RVEDVI (−0.29)	LAVI_max_ (−0.35)

*Numbers in parentheses are correlation coefficients.*

*RHC, right heart catheter; LV, left ventricle; RV, right ventricle; LA, left atrium; v_S_, systolic LA peak inflow velocity; v_D_, diastolic LA peak inflow velocity; v_E_, early diastolic LA peak outflow velocity; v_A_, late diastolic LA peak outflow velocity; mPAP, mean pulmonary arterial pressure; sPAP, systolic pulmonary arterial pressure; dPAP, diastolic pulmonary arterial pressure; PAWP, mean pulmonary artery wedge pressure; sBP, systolic systemic blood pressure; dBP, diastolic systemic blood pressure; EF ejection fraction; EDVI, normalized end-diastolic volume; ESVI, normalized end-systolic volume; SVI, normalized stroke volume; CI, cardiac index; LAVI_max_, normalized maximal left atrial volume*.

### Modeling of the LA acceleration factor

Assuming a linear relationship between PAWP and α_general_, regression of PAWP on *v*_E_, *v*_A_, *v*_S_ and *v*_D_ for all patients with biphasic transmitral flow profile (*n* = 56) resulted in a non-linear correlation coefficient *r* = 0.94 and 95%-confidence intervals 0.53–1.14 and −0.06 to 0.33 for the constants *c*_out_ and *c*_in_, respectively. Fixing *c*_out_ = 1 and performing regression of PAWP on *v*_E_, *v*_S_ and *v*_D_ for all patients resulted in *r* = 0.94 and the 95%-confidence interval −0.05 to 0.26 for *c*_in_. Consequently, *c*_out_ = 1 and *c*_in_ = 0 or, equivalently, omission of *v*_A_- and *v*_S−D_-terms in α were optimal when searching for a linear correlate to PAWP. Notably *c*_in_ = 1 (simplifying α_general_ to *v*_E_/*v*_S_) was not in the derived 95%-confidence intervals, although *v*_E_/*v*_S_ correlated strongly with PAWP (*r* = 0.85).

### Relationship between LA acceleration factor and PAWP

Mean α for all patients was 1.69 ± 0.57. While mean α for patients without PH (1.46 ± 0.42) and for patients with pre-capillary PH (1.51 ± 0.40) did not differ significantly, both differed significantly from mean α in the post-capillary PH group (2.45 ± 0.41, *p* < 0.0001).

**Figure 3:**
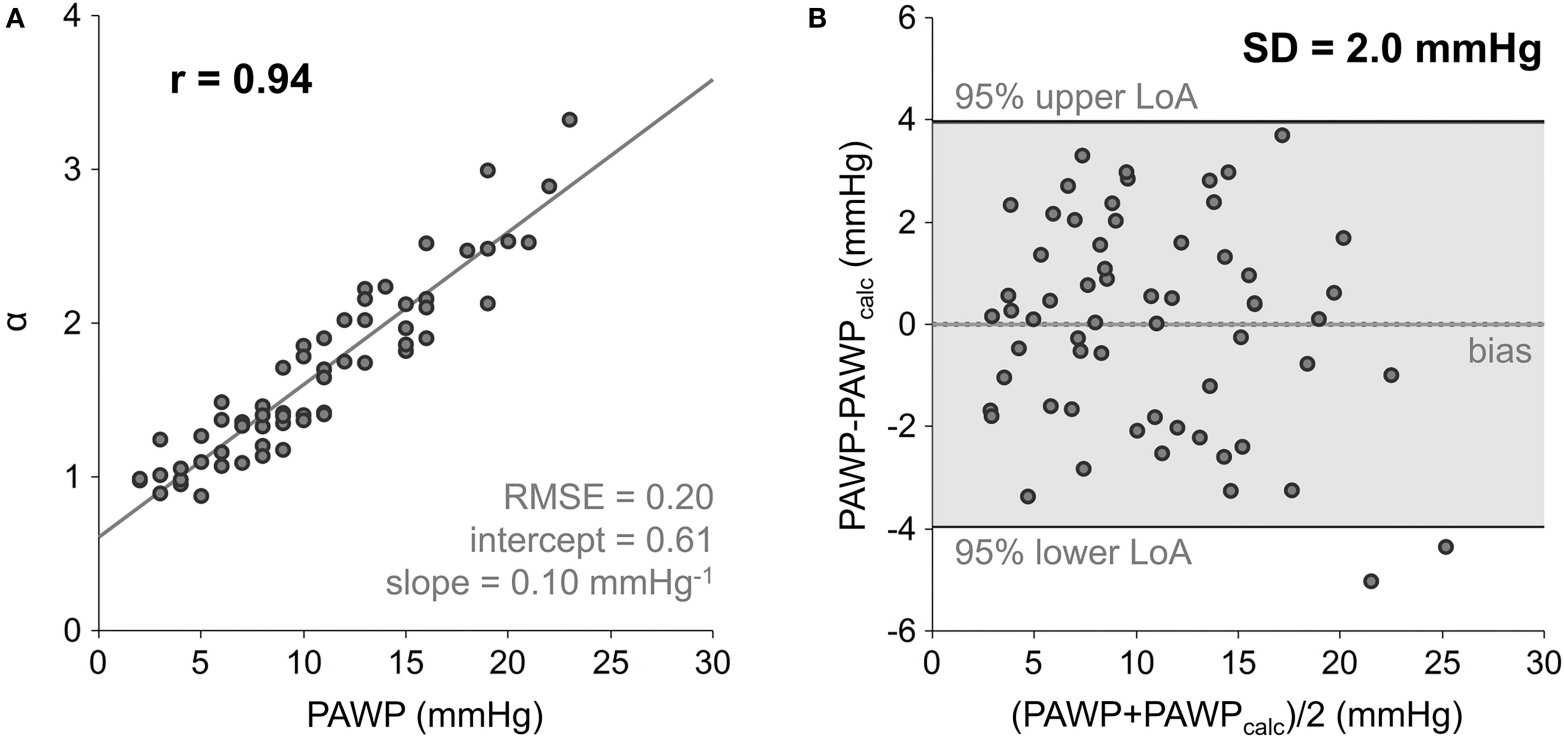
Scatter plot and linear regression of LA acceleration factor α on PAWP **(A)**, and Bland-Altman plot comparing RHC-derived PAWP and PAWP_calc_ calculated from inverted linear regression equation **(B)**. PAWP, mean pulmonary artery wedge pressure; RMSE, root-mean-square error; LoA, limits of agreement; SD, standard deviation of differences.

α correlated very strongly with PAWP (*r* = 0.94), even when restricted to the non-PH (*r* = 0.90), the pre-capillary PH (*r* = 0.91) and the post-capillary PH (*r* = 0.80) groups. The linear regression equation α = 0.61 + 0.10·PAWP (PAWP in mmHg) is shown in Figure 3A; the difference between *r*^2^ and prediction sum of squares (PRESS) *R*^2^ was small (0.01). Inversion of the linear regression equation to calculate PAWP from α (PAWP_calc_ = −6.2 + 10.1·α, with PAWP_calc_ in mmHg) resulted in a standard deviation of differences of SD = 2.0 mmHg between PAWP and PAWP_calc_; the corresponding Bland-Altman plot is presented in Figure 3B.

Neither intercept nor slope of the linear regression of α on PAWP depended significantly on gender, the presence of PH, or the evaluated pulmonary vein. Moreover, none of the continuous demographic, RHC and volumetric cardiac parameters assessed was a significant predictor of α additional to PAWP.

### Diagnosis of PAWP > 15 mmHg

**Figure 4:**
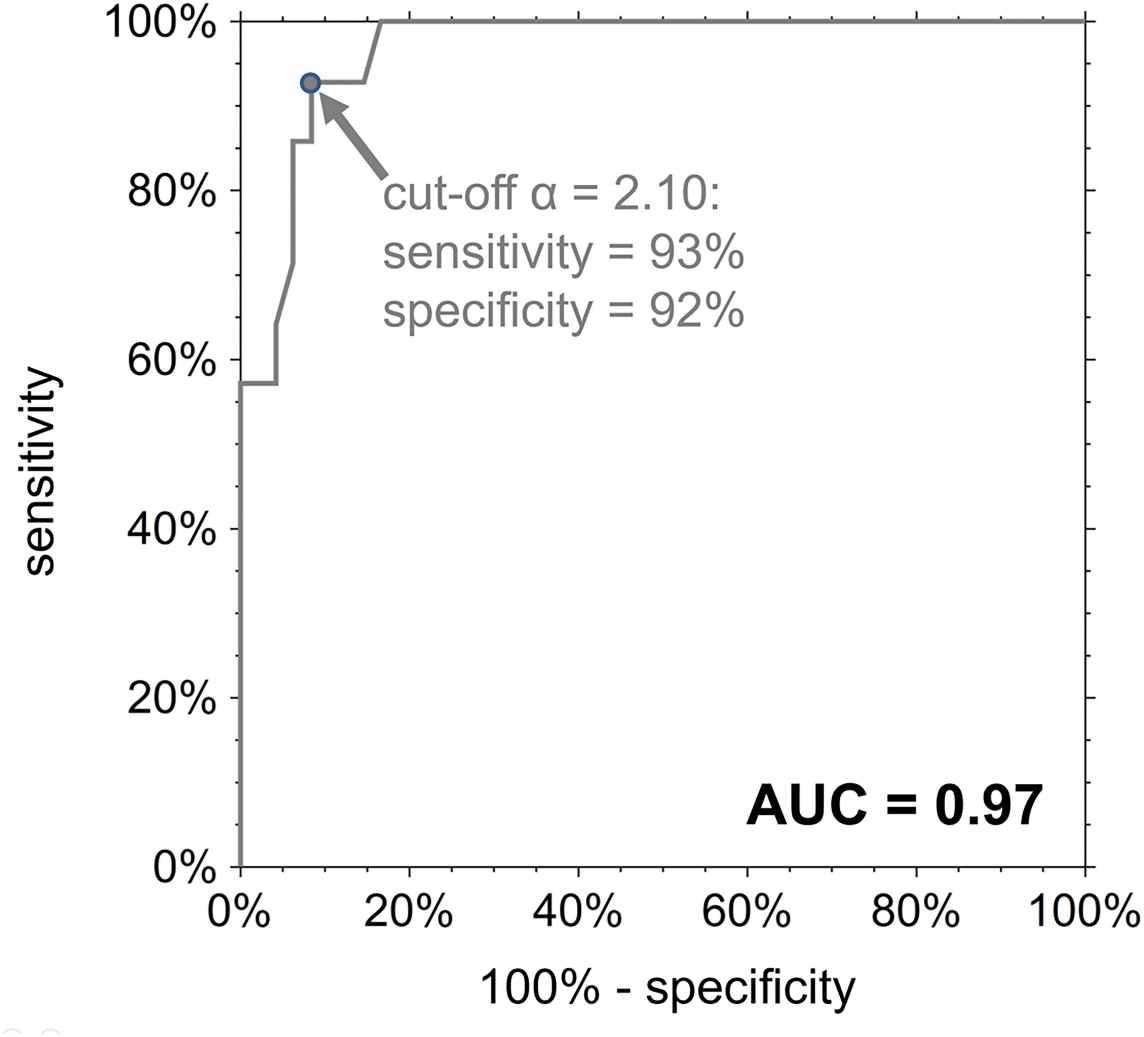
ROC curve for the diagnosis of PAWP > 15 mmHg employing the LA acceleration factor α. AUC, area under the ROC curve.

The area under the ROC curve (AUC) for the prediction of PAWP > 15 mmHg employing α was 0.97 with a 95%-confidence interval of 0.90–0.99 (Figure 4). The cut-off value α = 2.10 derived from the regression equation for PAWP = 15 mmHg coincided with the cut-off value maximizing the sum of sensitivity and specificity; the corresponding sensitivity and specificity were 93 and 92%, with 95%-confidence intervals of 66–100 and 80–98%, respectively.

The AUC for the diagnosis of post-capillary PH in PH patients was 0.95 with the 95%-confidence interval 0.83–0.99. Sensitivity and specificity for the cut-off value α = 2.10 were 92 and 86%, with 95%-confidence intervals of 64–100 and 64–97%, respectively, whereas the sum of sensitivity and specificity was maximized for the cut-off value α = 1.90 with sensitivity and specificity of 100 and 86% (95%-confidence intervals 75–100 and 64–97%), respectively.

### Locations of velocity measurements

Table 4 summarizes the results for LA peak in- and outflow velocities as well as derived acceleration factors determined from measurement planes at the level of mitral valve tips and in the pulmonary veins. The early diastolic transmitral peak velocity *v*_E, tip_ was the only velocity exhibiting a significant bias to *v*_E_. *v*_D, vein_ correlated, however, only weakly with *v*_D_. In contrast to measurements at the atrial junction, the means of *v*_D, vein_ in the non-PH (30 ± 8 cm·s^−1^), the pre-capillary PH (37 ± 8 cm·s^−1^) and the post-capillary PH (40 ± 12 cm·s^−1^) groups differed significantly between non-PH and PH subjects (*p* = 0.0026). Moreover, *v*_D, vein_ correlated significantly with sPAP (*r* = 0.44), mPAP (*r* = 0.33), dPAP (*r* = 0.31), PVR (*r* = 0.31), and TPG (*r* = 0.27).

**Table 4:** Peak velocities in the pulmonary vein and at the level of mitral valve tips and the corresponding LA acceleration factors.

**Parameter**	**Absolute**	***r***	**Bias**	***p*-value**
*v*_S, vein_ (cm·s^−1^)	35 ± 11	0.73	1 ± 9	0.4104
*v*_D, vein_ (cm·s^−1^)	34 ± 10	0.44	1 ± 10	0.2591
*v*_E, tip_ (cm·s^−1^)	60 ± 19	0.88	6 ± 9	<0.0001
*v*_A, tip_ (cm·s^−1^)	40 ± 17	0.81	2 ± 10	0.1704
α_tip_	1.88 ± 0.60	0.87	0.20 ± 0.30	<0.0001
α_vein_	1.63 ± 0.64	0.76	−0.05 ± 0.42	0.3251
α_tip/vein_	1.82 ± 0.68	0.65	0.13 ± 0.53	0.0527

*Absolute values and bias to the corresponding quantity determined at the atrial junction of the pulmonary vein and/or the atrio-ventricular junction are given as means ± standard deviations. r is the correlation coefficient between corresponding parameters determined at different locations. p-value refers to the significance test of the bias. v_S, vein_, systolic peak velocity determined in the pulmonary vein; v_D, vein_, diastolic peak velocity determined in the pulmonary vein; v_E, tip_, early diastolic peak velocity determined at the level of the mitral valve tips; v_A, tip_, late diastolic peak velocity determined at the level of the mitral valve tips; α_tip_, LA acceleration factor with early diastolic peak velocity determined at the level of the mitral valve tips; α_vein_, LA acceleration factor with peak velocities determined in the pulmonary vein; α_tip/vein_, LA acceleration factor with peak velocities determined in the pulmonary vein and at the level of the mitral valve tips, respectively*.

**Figure 5:**
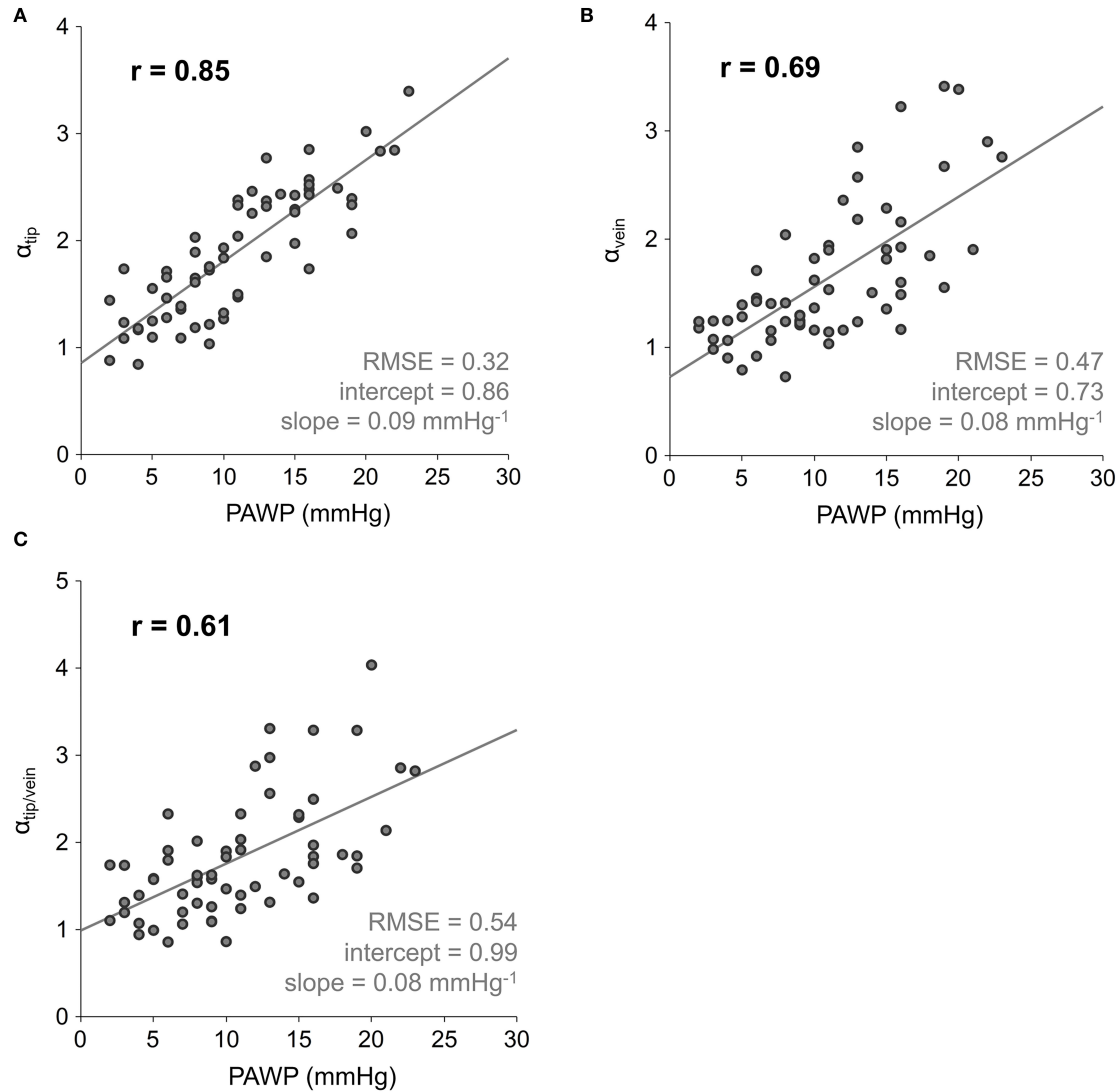
Scatter plots and linear regressions of the LA acceleration factors on PAWP, with LA acceleration factors calculated from peak velocity measurements at the level of mitral valve tips α_tip_ **(A)**, within the pulmonary vein α_vein_ **(B)**, and both α_tip/vein_ **(C)**. PAWP, mean pulmonary artery wedge pressure; r, correlation coefficient; RMSE, root-mean-square error.

Linear regressions of α_tip_, α_vein_ and α_tip/vein_ on PAWP are shown in Figure 5. Compared to the correlation between PAWP and α, all correlations between PAWP and LA acceleration factors derived from peak velocity measurements at the mitral valve tips and in the pulmonary veins were significantly weaker (*r* = 0.85, *p* = 0.0004 for the correlation between PAWP and α_tip_, *r* = 0.69, *p* < 0.0001 for the correlation between PAWP and α_vein_, and *r* = 0.61, *p* < 0.0001 for the correlation between PAWP and α_tip/vein_). Notably, intercepts of the linear regression lines of α_vein_ and α_tip/vein_ on PAWP depended significantly on the presence of PH (*p* = 0.6844, 0.0012, and 0.0094 for the linear regressions of α_tip_, α_vein_ and α_tip/vein_ on PAWP, respectively).

## Discussion

The main results of the study were that the LA acceleration factor α (1) represented an optimal linear correlate to PAWP among all ratios of linear combination of *v*_E_ and *v*_A_ to linear combinations of *v*_S_ and *v*_D_, (2) correlated very strongly with PAWP, (3) allowed an accurate prediction of PAWP > 15 mmHg, and (4) depended significantly on the location of measurement of *v*_E_, *v*_S_ and *v*_D_.

### The LA acceleration factor

Dependencies of 4D flow-derived LA peak in- and outflow velocities on PAWP, specifically an increase of *v*_E_, a decrease of *v*_S_ and non-significant changes of *v*_A_ and *v*_D_ with PAWP, are in line with the common interpretation of mean LA pressure as a major determinant of pulmonary venous and early diastolic transmitral blood flow velocities (14–16). A similar relationship of echocardiographically determined transmitral peak velocities to PAWP was found previously in populations including both, pre- and post-capillary PH patients (17–20). The dependency of pulmonary venous peak inflow velocities on PAWP rather than on TPG, which was similarly shown in an animal model study (21), justifies our hypothesis that the LA acceleration factor would correlate with PAWP.

Given the dependencies of LA peak in- and outflow velocities on PAWP and the observed independence of *v*_S_ and *v*_E_, one might have expected the LA acceleration factor correlating with PAWP to be of the form *v*_E_/*v*_S_. Regression of PAWP on *v*_E_, *v*_A_, *v*_S_ and *v*_D_ suggested, however, that α takes the form *v*_E_/*v*_S+D_ with *v*_S+D_ being the average of *v*_S_ and *v*_D_. This functional form of α did not particularly depend on PH and its pre- or post-capillary nature, because α and PAWP correlated very strongly, even when restricted to the non-PH, the pre-capillary PH or the post-capillary PH groups with their smaller PAWP ranges.

Two arguments that are not mutually exclusive support the appearance of *v*_D_ in the definition of α: First, maximal early diastolic transmitral blood flow velocity depends on left ventricular relaxation rate (14–16). In a close correlate between PAWP and an expression containing *v*_E_, this dependency should be corrected. *v*_D_ correlated significantly with *v*_E_, and the early diastolic pulmonary venous peak velocity tends to share dependencies with early diastolic transmitral blood velocity (14–16). Therefore, *v*_D_ in α might be interpreted as a correction term for left ventricular relaxation. Second, velocities and kinetic energy of blood within the LA in early diastole should not only depend on systolic inflow characterized by *v*_S_ but should increase and decrease with the early diastolic pulmonary venous inflow characterized by *v*_D_. Higher or lower kinetic energy and velocities in the LA in early diastole imply smaller or higher LA pressures necessary to accelerate the blood to a specific transmitral peak velocity *v*_E_, such that the appearance of *v*_D_ in α might be interpreted as “input velocity correction” from which acceleration by LA pressure or PAWP occurs.

### The relationship between LA acceleration factor and PAWP

The very strong correlation between α and PAWP suggests that α can be employed to estimate PAWP from 4D flow measurements. Inverting the linear regression equation of α on PAWP, the standard deviation of errors between non-invasively estimated and RHC-derived PAWP was substantially smaller than standard deviations found when employing either the quotient of early diastolic transmitral peak blood flow velocity to mitral annular peak tissue velocity (22–24) or LA volume and strain parameters (25, 26) for estimation of PAWP in PH. Notably, the correlation between LAVI_max_ and PAWP in the present study population was similar to the one previously found by Swift et al. (25).

The accuracy of α for discriminating between subjects with PAWP > 15 mmHg and PAWP ≤ 15 mmHg exceeded accuracies of previously studied single-parametric models (17–20, 22, 23, 25–29) and multi-parametric models (19, 20, 30, 31).

As expected by known direct or indirect relationships of forward peak velocities of pulmonary venous and transmitral flow profiles on age, systemic blood pressure, LV, RV and LA volumes, and on LV as well as RV systolic performance (14–16, 32), *v*_E_, *v*_A_, *v*_S_ and *v*_D_ demonstrated various weak to moderate univariate associations with these parameters in the present study population. The linear regression equation of α on PAWP, however, exhibited neither significant dependency on any of these demographic or cardiac volumetric parameters nor on any RHC-derived parameters apart from PAWP. This fact, together with the high PRESS R^2^, suggest an adequate generalizability of the predictive results for the functional relationship between α and PAWP in PH patients (33, 34).

### Locations of velocity measurements

While it is canonical to determine maximal 3D LA in- and outflow velocities at the atrial junctions of the pulmonary veins and atrio-ventricular junction with MR 4D flow data, MR 2D phase contrast (35) or echocardiography (16) derived transmitral and pulmonary venous flow profiles are typically assessed at the level of mitral valve tips and 1–2 cm in the pulmonary veins, respectively. Employing 3D peak velocities measured at these locations worsened the correlations of LA acceleration factors with PAWP. This result might be immediately understood as reflecting the fact that peak velocities in the pulmonary veins and at the level of the mitral valve tips contain dependencies on geometrical factors such as the mitral valve's opening area or the pulmonary vein's cross section and curvature (14, 36), which are not directly related to mean LA pressure or PAWP. Moreover, the presence of PH explained a significant part of the variability of the linear relationship between PAWP and α_vein_ or α_tip/vein_, which might be attributed to the fact that early diastolic peak blood flow velocity in the pulmonary veins depended on pulmonary artery pressures.

### Limitations

Some limitations of the current study need to be acknowledged. Although the delay between RHC and MR imaging was small, measurements were not acquired simultaneously. Typical differences between measured PAWP and PAWP estimated from MR imaging should amount to at least 9% (37), but it seems legitimate to assume that the delay only contributes to worsening of correlations. Additionally, different procedures for invasive PAWP measurement (38, 39) might alter its relationship with α.

Linear regression of LA acceleration factor on PAWP as well as Bland-Altman plot comparing RHC-derived PAWP and calculated PAWP were derived from the whole study population, because PRESS statistics rather than data splitting was employed for cross-validation.

The assumption of a linear relationship between LA acceleration factor and PAWP was well fulfilled within the relatively small range of PAWP values of the patients in our study. While a larger PAWP range might have increased the correlation, deviations from linearity might occur in populations with very high PAWP values.

Finally, the time resolution and slice thickness of the phase-contrast sequence used, as well as the coverage of the LA, were optimized for total imaging time. The limited spatiotemporal resolution might have caused underestimation of the true peak velocities. Because LA in- and outflow waves do not change rapidly with time and peak velocities compared well with those in studies employing 4D flow technique with higher spatial and/or temporal resolution (8, 9, 13, 40), one could expect the impact of resolution to be moderate, especially for the estimation of α as a quotient of velocities. The incomplete coverage of the LA and all entering pulmonary veins did not pose a direct limitation to the results. However, neither the inflow via the upper pulmonary veins nor the propagation of maximal velocities inside the LA could be investigated adequately in the studied population.

## Conclusions

The LA acceleration factor α was introduced as the 4D flow-derived ratio of early diastolic LA peak outflow velocity at the atrio-ventricular junction to the average of systolic and early diastolic LA peak inflow velocities at the atrial junctions of the pulmonary veins. The very close relationship between α and RHC-derived PAWP suggests α as potential non-invasive parameter for the estimation of PAWP and the distinction between pre- and post-capillary PH patients.

## Data availability statement

The datasets used and analyzed during the current study are available from the corresponding author on reasonable request.

## Ethics statement

The study was approved by the local ethical review board and all subjects gave written informed consent.

## Author contributions

GR: study design, data acquisition, statistical analysis, manuscript preparation, and editing. GK: study design, data acquisition, and manuscript editing. CR: study design, data analysis, and manuscript editing. AS, MF, and HO: study design and manuscript editing. UR: study design, data acquisition and analysis, manuscript preparation, and editing. All authors read and approved the final manuscript.

## Funding

This work was supported by the funds of the Oesterreichische Nationalbank, Anniversary Fund (Grant Number 15702).

## Conflict of interest

Author GR is an employee of Siemens Healthcare Diagnostics GmbH. The remaining authors declare that the research was conducted in the absence of any commercial or financial relationships that could be construed as a potential conflict of interest.

## Publisher's note

All claims expressed in this article are solely those of the authors and do not necessarily represent those of their affiliated organizations, or those of the publisher, the editors and the reviewers. Any product that may be evaluated in this article, or claim that may be made by its manufacturer, is not guaranteed or endorsed by the publisher.

## Abbreviations

2D: two-dimensional
3D: three-dimensional
4D: four-dimensional
ANOVA: analysis of variance
AUC: area under the receiver operating characteristic curve
α: left atrial acceleration factor
BSA: body surface area
bSSFP: balanced steady state free precession
CI: cardiac index
dBP: diastolic systemic blood pressure
dPAP: diastolic pulmonary arterial pressure
ECG: electrocardiogram
EDVI: normalized end-diastolic volume
EF: ejection fraction
ESVI: normalized end-systolic volume
LA: left atrium
LAVI_max_: maximal left atrial volume normalized to body surface area
LoA: limits of agreement
LV: left ventricle
MMI: normalized muscle mass
mPAP: mean pulmonary arterial pressure
MR: magnetic resonance
PAWP: mean pulmonary artery wedge pressure
PH: pulmonary hypertension
PV: pulmonary vein
PVR: pulmonary vascular resistance
PRESS: prediction sum of squares
*r*: Pearson's correlation coefficient
R: multiple non-linear correlation coefficient
RHC: right heart catheter
RMSE: root-mean-square error
ROC: receiver operating characteristic
RV: right ventricle
sBP: systolic systemic blood pressure
SD: standard deviation of differences
sPAP: systolic pulmonary arterial pressure
SVI: normalized stroke volume
TPG: transpulmonary pressure gradient
*v*_A_: late diastolic left atrial peak outflow velocity
*v*_D_: diastolic left atrial peak inflow velocity
*v*_E_: early diastolic left atrial peak outflow velocity
*v*_S_: systolic left atrial peak inflow velocity.
